# Impact of preoperative patient information on anxiety and physiological responses in mandibular third molar surgery: A randomized controlled trial

**DOI:** 10.4317/medoral.27904

**Published:** 2026-03-07

**Authors:** Ummugulsum Coskun, Nuray Yilmaz Altintas, Burak Cezairli, Kerem Turgut Atasoy

**Affiliations:** 1 Altinbas University Faculty of Dentistry, Department of Oral and Maxillofacial Surgery, 34147 Istanbul, Turkey; 2 Private Practice, Department of Oral and Maxillofacial Surgery, 34840 Istanbul, Turkey; 3 Private Practice, Department of Oral and Maxillofacial Surgery, 16270 Bursa, Turkey

## Abstract

**Background:**

This study aimed to investigate the impact of different patient information modalities on anxiety levels and physiological responses during the surgical extraction of impacted mandibular third molars.

**Material and Methods:**

A prospective randomized clinical trial was conducted involving 97 individuals undergoing surgical removal of impacted mandibular third molars. Participants were allocated into three groups: Control, verbal, and visual. Anxiety was measured using the Modified Dental Anxiety Scale (MDAS) and the State-Trait Anxiety Inventory (STAI). Heart rate, blood pressure, and oxygen saturation (SpO2) were recorded at five surgical stages. Pain was assessed using a Visual Analog Scale (VAS).

**Results:**

VAS scores did not differ between groups. Significant intergroup differences were observed in diastolic blood pressure after local anesthesia and in SpO2 during tooth extraction and postoperatively (p&lt;0.05). All groups showed reductions in MDAS, STAI-T, and STAI-S from the preoperative to the postoperative period. MDAS decreased significantly in the control and visual groups, while STAI-T decreased in all groups (p&lt;0.05). STAI-S showed no significant change. Trial registration: ClinicalTrials.gov Identifier: NCT07018115; Registration date: 10 June, 2025.

**Conclusions:**

Although no significant differences were found between information methods in reducing anxiety, providing preoperative information may enhance patient comfort during third molar surgery. Further studies are needed to determine the most effective strategies for patient education.

## Introduction

Anxiety is an unpleasant emotional reaction accompanied by activation of the autonomic nervous system in response to a perceived threat (1). Dental anxiety and phobias are common among patients and may impair oral health and complicate clinical procedures (2). This condition also poses a challenge for clinicians, resulting in treatment difficulties, increased procedural time, and reduced quality of care (3).

Third molar extraction is one of the most common surgical treatments in maxillofacial surgery. Anxiety may affects this procedure as well, leading to prolonged operation times and increased postoperative morbidity, including swelling and pain (4). Dental anxiety during third molar surgery may also result in hemodynamic changes such as elevated blood pressure and heart rate (5). Therefore, reducing preoperative anxiety is crucial for improving surgical outcomes (6). Studies suggest that improving patient knowledge may decrease preoperative anxiety and enhance comprehension (4 , 7). Patient education through written materials or verbal information is commonly used; however, written terminology may not always be clearly understood, and verbal explanations are often easily forgotten (8).

With advances in media technologies, visual information such as videos and computer animations may improve understanding and potentially reduce anxiety (9). However, studies have reported mixed results when comparing video-assisted education with verbal or written information (6 , 8). Clinically, some patients prefer not to receive detailed information, whereas others arrive after watching third molar extraction videos online. Therefore, this study aimed to determine whether different educational techniques, compared with a control group receiving only basic information, reduce anxiety in patients undergoing impacted third molar removal.

We hypothesized that educational techniques about the surgical procedure would improve patient understanding of surgery. However, detailed visual information about the surgical technique might increase anxiety related to third molar removal. The specific aim of this study was to compare patient anxiety levels between the treatment and control groups using anxiety scales and by measuring the physiological indicators such as heart rate (HR), systolic blood pressure (SBP), diastolic blood pressure (DBP), and oxygen saturation (SpO).

## Material and Methods

Study Design/Sample

To address the specific aim, a prospective randomized clinical trial was conducted. The study sample consisted of patients who were admitted to the Department of Oral and Maxillofacial Surgery, Faculty of Dentistry, Karadeniz Technical University, for third molar extraction. The study protocol was approved by the Ethics Committee of Karadeniz Technical University (2015/30) and was conducted in accordance with the Declaration of Helsinki. Written informed consent was obtained from all participants. In addition, all patients received a standard institutional consent form including information about the procedure, potential risks, benefits, and postoperative care instructions.

Sample size calculation was performed a priori using G*Power software, based on the primary anxiety outcome (10). The calculation was informed by a previously published randomized clinical trial (11). With an alpha level of 0.05 and a statistical power of 95%, the minimum required sample size was determined to be 28 participants per group. To account for potential data loss, a total of 105 participants were included.

**Figure 1:**
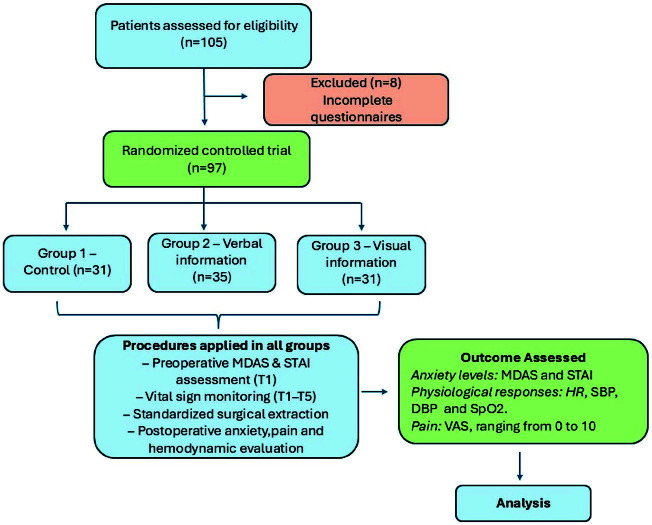
Study flow diagram illustrating the screening, exclusion, group allocation, interventions, and outcome assessment steps in the randomized controlled trial.

Eligible participants were adults (18 years) with impacted third molars who were able to read and complete the questionnaires and agreed to participate. Exclusion criteria included age &lt;18 years, systemic diseases contraindicating surgery, poor oral hygiene, pericoronitis, and pregnancy. All extractions were performed under local anesthesia without pharmaceutical premedication or intravenous sedation. The overall study design and participant flow are shown in Figure 1.

1

Study Variables

The predictor variable was the type of preoperative information, consisting of three groups: A control group, a verbally informed group, and a visually informed group. Each group was further subdivided into male and female subjects. Participants were randomly assigned to the three groups. In the control group (Group 1), patients received only a brief verbal explanation of the procedure. In Group 2, patients received a detailed verbal explanation of each surgical step using non-technical language. In Group 3, the same verbal explanation was supplemented with 2-D animated visual illustrations (anesthesia, incision, possible osteotomy or tooth sectioning, and suturing). All patients were additionally informed about expected numbness after anesthesia, vibration sensation from the bur, and painless pressure during surgical steps.

The primary outcomes of the study were anxiety levels and physiological responses. Anxiety was assessed using the Modified Dental Anxiety Scale (MDAS) and the State-Trait Anxiety Inventory (STAI), while physiological responses included HR, SBP, DBP, and SpO, which were measured repeatedly at predefined surgical time points.

The secondary outcome was intraoperative pain, assessed using a Visual Analog Scale (VAS) ranging from 0 (no pain) to 10 (severe pain).

A dental anxiety score 19 on the MDAS was considered high anxiety, as previously validated in the literature (12). Additional data collected included age, gender, education, and the anatomical position of the treated third molar.

Data Collection and Practical Procedure

Two surgeons performed all procedures following a standardized technique to minimize operator variability. Participants were randomly assigned to one of the three study groups using a computer-generated randomization sequence created in Microsoft Excel. Group allocation was revealed sequentially as each patient arrived, and the operating surgeon learned the assigned group only at the time of the procedure. Outcome evaluators were blinded to group allocation. The researcher responsible for measuring and recording vital signs at each surgical time point did not know which information modality had been presented to the participant.

On the day of treatment, eligible patients completed the STAI and MDAS scales in the waiting room before receiving any information. The STAI consists of 40 items (20 assessing trait anxiety and 20 assessing state anxiety) scored from 20 to 80, while the MDAS includes 5 items scored 1-5 (13). Demographic data were recorded, and hemodynamic parameters (HR, SBP, DBP, SpO) were measured at five time points: Before surgery (T1), after information presentation and before local anesthesia (T2), after local anesthesia (T3), during extraction (T4), and postoperatively (T5).

Impaction depth was classified radiographically as Level A, B, or C according to the vertical position of the third molar, similar to the Pell and Gregory system (14). Winter's classification was used to identify tooth angulation, and the relationship to the ramus was also recorded.

All physiological measurements (SBP, DBP, HR, and SpO) were obtained under standardized and identical conditions for all participants. Vital signs were recorded using the same digital monitoring device, with patients positioned in the same seated posture, and in the same operatory environment. Measurements were performed at the same predefined surgical time points for every participant. This protocol ensured uniformity in both timing and measurement conditions across the study and minimized potential measurement bias.

All extractions were carried out under local anesthesia without pharmaceutical premedication or intravenous sedation. After anesthesia administration, the impacted tooth was removed using incision, flap reflection, osteotomy and, if necessary, tooth sectioning. The socket was irrigated, debrided, and closed using 4-0 silk sutures. A researcher recorded hemodynamic values at each surgical stage.

Data Analysis

Descriptive statistics were reported as mean ± standard deviation, median, and range. Data distribution was evaluated using the Shapiro-Wilk test, and variance homogeneity was assessed with Levene's test. When the assumption of normality was met, the Independent Samples t-test was used for comparisons between two independent groups, and one-way ANOVA was applied for comparisons among three groups. For normally distributed data, the Paired Samples t-test was used for comparisons between two dependent groups. When the assumption of normality was not met, the Friedman test was used to examine differences among three dependent groups. Post hoc Bonferroni and adjusted Bonferroni tests were applied to identify the group or groups responsible for significant differences. Pearson correlation analysis was used to examine relationships between normally distributed continuous variables, whereas Spearman correlation analysis was applied for relationships involving ordinal categorical variables and continuous variables. Pearson's chi-square test was used to examine associations between categorical variables when the sample size assumption (expected cell count &gt;5) was met. All analyses were performed using the IBM Statistical Package for Social Sciences (SPSS) for Windows, version 27 (SPSS Inc., Chicago, IL, USA).

## Results

A total of 105 patients met the eligibility criteria; however, 8 were excluded due to incomplete questionnaire data, resulting in 97 patients for analysis. All underwent unilateral mandibular impacted third molar extraction with tooth sectioning and bone removal. Local anesthesia was administered using 2mL Ultracain D-S (4% articaine with epinephrine 1:200,000; Aventis, The Netherlands).

Demographic Data

The study sample consisted of 97 patients (mean age: 24.50±5.83 years), including 44 women (25.31±6.86 years) and 53 men (23.83±4.77 years). Demographic variables (age, gender, education level, previous dental experience, smoking status, and systemic disease) were similar across groups, with no statistically significant differences. Although the distribution of education level did not differ significantly between groups, most participants had a high school or associate degree.

Impacted Third Molars

Each patient underwent extraction of one mandibular impacted third molar. The distribution of tooth positions and ramus relationships was similar across groups. While bone level C did not differ significantly, Bone Level A was significantly more common in Group 1 and Bone Level B was significantly more common in Group 2 (p&lt;0.05). Conversely, Bone Level A was significantly lower in Group 2 and Bone Level B was significantly lower in Group 1 (p&lt;0.05) (Table 1). Exploratory analyses showed no clinically or statistically meaningful association between Bone Level A/B classification and anxiety scores (MDAS, STAI), physiological parameters, or intraoperative pain (VAS) (p&gt;0.05).

Table 1VAS

There were no significant differences in VAS scores among the groups. The mean scores for the groups were 0.35±0.87) for Group 1,0.60±1.09) for Group 2 and 0.67±1.24) for Group 3.

Operation Duration

A statistically significant difference in operation duration was observed among the groups (p&lt;0.05). Group 1 had longer operation times than the other groups. Mean operation time was 18.03±11.86 minutes in Group 1, 11.88±5.71 minutes in Group 2, and 12.09±5.88 minutes in Group 3.

Vital Signs

**Figure 2:**
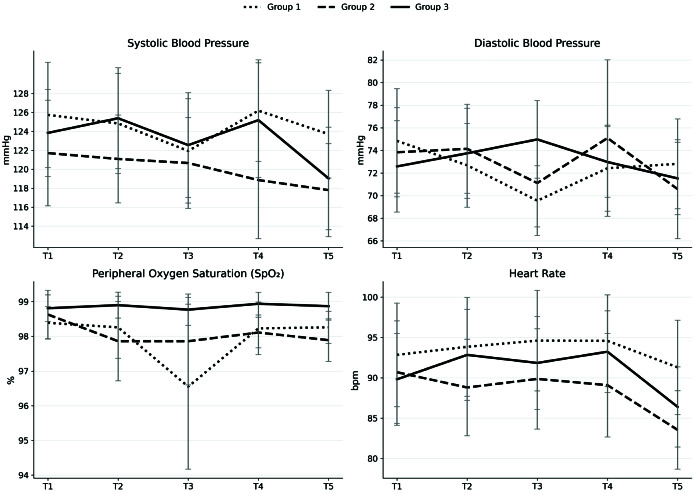
Longitudinal line graph of vital signs for three groups. *Significant differences were observed in SpO2 (T4), SpO2 (T5) and DBP (T3).

The results of the intergroup comparisons are summarized in Table2. A statistically significant difference was observed in DBP at T3 among the groups (p&lt;0.05). Figure 2 illustrates the longitudinal changes in vital signs, showing significant differences in SpO (T4), SpO (T5) and DBP (T3) among the groups. The T3 DBP values were similar in Groups1 and2, with the difference attributable to Group3. Similarly, a statistically significant difference was found for the T4 SpO measurement (p&lt;0.05), with comparable values in Groups1 and2, and the difference again originating from Group3.

Table 2 2

Furthermore, the T5 SpO measurement showed a statistically significant difference between groups (p&lt;0.05), where Groups1 and3 had similar values, and the difference was attributable to Group2. In contrast, no statistically significant intergroup differences were detected for any of the remaining measurements (p&gt;0.05), indicating that these parameters were not influenced by group allocation.

Anxiety Tests

All groups demonstrated reductions in MDAS, STAI-T and STAI-S scores from the preoperative to the postoperative assessments. However, no statistically significant intergroup differences were observed in preoperative or postoperative MDAS, STAI-T, or STAI-S scores (Table 3). Within-group analysis showed significant decreases in MDAS scores in Groups 1 and 3 (p&lt;0.05), and significant decreases in STAI-T in all three groups (p&lt;0.05). No significant pre-post change was detected in STAI-S in any group. Subgroup analyses revealed that improvements were more apparent in male patients than in females across all groups.

Table 3

## Discussion

In the digital era, patients frequently access online information and videos related to dental procedures, which may either increase or decrease preoperative anxiety depending on the content and individual characteristics. The present study aimed to investigate whether different preoperative information modalities influence anxiety levels in patients undergoing impacted third molar extraction.

Consistent with the literature, anxiety tends to peak before surgery and decrease following treatment (15 - 17). In our study, although physiological parameters such as HR, SBP, and DBP decreased postoperatively, significant intergroup differences were limited to SpO levels at T4 and T5. HR levels in the control group were consistently higher than those in the other groups at all time points. Overall, physiological responses were not strongly influenced by the information modality provided.

Pain and anxiety can directly increase heart rate by stimulating the adrenal medulla, and physiological measurements are considered more reliable indicators of anxiety than self-reported scales (15). Although the graphical trends of vital signs varied among groups, no statistically significant intergroup differences were observed in this study. Previous studies have shown that the most pronounced cardiovascular changes occur before local anesthesia administration and during tooth extraction (18 , 19). However, in our study, the most pronounced change in heart rate was observed during the postoperative period, although this difference was not statistically significant. This finding may be related by the persistence of elevated catecholamine levels after surgery, which could lead to delayed cardiovascular responses in some patients.

Sharma et al. (18) reported significant increases in HR and SBP/DBP during local anesthetic administration in patients with high dental anxiety, while Alemany-Martínez et al. (6) found the lowest HR values before surgery and the highest values during incision and flap reflection. Similarly, Gadve et al. (20) observed increased HR and DBP during anesthesia and extraction, suggesting that endogenous catecholamine release due to emotional stress, rather than pharmacologic effects, primarily drives these hemodynamic changes. These findings indicate that dental anxiety can substantially influence physiological responses during the most stressful surgical phases. In our study, although no statistically significant difference was observed, heart rate increased after anesthesia administration, while the lowest value was recorded at the end of the procedure. This hemodynamic parameter has been mentioned in previous studies as a reliable method for assessing stress levels during dental procedures (21 , 22). However, it should be noted that changes in blood pressure and heart rate may also be influenced by individual factors such as age, gender, current medications, the use of local anesthetics containing adrenaline, and previous dental experiences (23).

Saincher et al. (23) reported higher preoperative SBP and DBP and higher SpO values in the video group during incision and drilling, consistent with stress-related physiological responses. Similarly, in our study, SpO at T4 was significantly higher in the animation group than in the verbally informed group, suggesting that animation-based information may help maintain physiological stability during surgery by reducing intraoperative anxiety or enhancing patient preparedness.

In our study, the mean intraoperative VAS scores recorded were low in all groups, which may partly explain the absence of significant differences in postoperative anxiety levels. Although Bone Level A and B distributions differed significantly between groups, these variations did not influence postoperative pain or anxiety outcomes. This suggests that the degree of impaction alone may not be a major determinant of patient stress or discomfort during third molar surgery.

Akomolafe et al. (24) reported that verbal information provided before surgery reduced anxiety more effectively than audiovisual information in third molar patients. Similarly, Erbasar et al. (25) found that non-voiced visual materials and written brochures reduced anxiety more than verbal communication alone. In agreement with these studies, our findings also showed that animation-based visual information without voiceover contributed to lower intraoperative anxiety indicators such as reflected by higher SpO levels. Silent visual materials may allow patients to process information more calmly without overstimulation.

In the present study, most patients exhibited high preoperative anxiety levels. Although both STAI-T and STAI-S scores decreased postoperatively, only STAI-T showed a statistically significant reduction. Kazancolu et al. (8) similarly reported a significant decrease in STAI-T, while STAI-S also improved in their study. Kakinuma et al. (26) found that animation-based information enhanced patient understanding without reducing anxiety, which is consistent with our results. Hollander et al. (15) reported lower MDAS scores in males; however, in our study, males had higher MDAS and STAI scores, although the differences were not statistically significant. This discrepancy may reflect the multifactorial nature of dental anxiety, which may be influenced by age, education, dental history, and treatment expectations, rather than gender alone.

Providing information about the operative procedure has been reported to reduce patient uncertainty (15). In our study, the control group showed higher preoperative STAI-T and STAI-S scores (p&gt;0.05), and both verbal and visual information tended to reduce anxiety, although not at a statistically significant level. The absence of significant differences between groups may be related to the relatively high educational level and awareness of most participants, which could have attenuated the impact of the information modality on anxiety reduction.

Although verbal and visual information modalities did not produce statistically significant reductions in self-reported anxiety scores, animation-based education was associated with more stable physiological responses, particularly among female patients. These findings suggest that multimedia tools may enhance patient comfort and physiological preparedness during oral surgical procedures. Incorporating visual information into standard preoperative protocols may therefore be beneficial, especially in patients with high baseline anxiety or limited dental experience. Clinically, these results support the integration of tailored animation-based materials to improve patient cooperation and overall surgical experience.

This study has several limitations. It was conducted in a single academic center with a relatively young and systemically healthy population, which may limit the generalizability of the findings to older individuals, patients with systemic comorbidities, or those with high baseline anxiety. Although Bone Level A/B distribution differed across groups, exploratory analyses did not indicate a clinically or statistically meaningful influence on anxiety, physiological parameters, or intraoperative pain; however, residual confounding related to unequal impaction depth distribution cannot be completely excluded. In addition, physiological responses may have been affected by unmeasured factors such as caffeine intake, baseline stress levels, or individual pain thresholds. Finally, anxiety and physiological outcomes were assessed only during the immediate perioperative period, without 24-72-hour follow-up and patient-reported experience measures, limiting the evaluation of early recovery. Future multicenter trials with larger and more diverse samples, extended postoperative follow-up, and evaluations of alternative digital information methods are needed to enhance the external validity and clinical relevance of these findings.

## Conclusions

Anxiety associated with third molar surgery is multifactorial and cannot be explained by a single determinant. In this study, both verbal and animation-based preoperative information appeared to support reductions in anxiety and improve patient understanding; however, the lack of significant differences in anxiety scores and hemodynamic changes between the groups suggests that these modalities alone may not be sufficient. Animation-based tools may still contribute positively to patient experience, but further research is needed to identify alternative or combined educational approaches that more effectively reduce dental anxiety in clinical practice.

## Figures and Tables

**Table 1:** Baseline demographic and clinical characteristics of patients in the control, verbal, and visual information groups.

Variable	Groups		
	Control (n=31)	Verbal (n=35)	Visual (n=31)
Age			
Gender (Female/Male)	24.83 (±5.73)	25.11 (±6.50)	23.48 (±5.15)
Education	15/16	18/17	Nov-20
Illiterate			
Primary School	1 (3.23%)	2 (5.71%)	1 (3.22%)
High School	10 (32.26%)	7 (20.00%)	9 (29.03%)
Associate Degree	3 (9.68%)	4 (11.43%)	3 (9.67%)
Graduate degree or above	17 (54.84%)	21 (60.00%)	16 (51.61%)
Experience	0 (0.00%)	1 (2.86%)	2 (6.45%)
Bad experience	11 (35.48%)	10 (28.57%)	10 (32.25%)
Smoking	2 (6.45%)	1 (2.86%)	1 (3.22%)
Alcohol	9 (29.03%)	10 (28.57%)	4 (12.90%)
Systematic Disease	2 (6.45%)	5 (14.29%)	0 (0.00%)*
Drug	5 (16.13%)	3 (8.57%)	4 (12.90%)
Complaint	3 (9.68%)	5 (14.29%)	3 (9.67%)
Impacted Tooth	21 (67.74%)	23 (65.71%)	23 (74.19%)
Position			
Mesioangular	10 (32.26%)	6 (17.14%)	16 (51.61%)
Vertical	2 (6.45%)	5 (14.29%)	2 (6.45%)
Horizontal	18 (58.06%)	23 (65.71%)	12 (38.70%)
Distoangular	1 (3.23%)	1 (2.86%)	1 (3.22%)
Bone level			
Level A	12 (38.71%)	20 (57.14%)*	7 (22.58%)*
Level B	10 (32.26%)	7 (20.00%)*	20 (64.51%)*
Level C	9 (29.03%)	8 (22.86%)	4 (12.90%)
Distance to Ramus			
Class I	9 (29.03%)	6 (17.14%)	3 (9.67%)
Class II	18 (58.06%)	20 (57.14%)	22 (70.96%)
Class III	4 (12.90%)	9 (25.71%)	6 (19.35%)
			
VAS (0-10)	0.35 (±0.87)	0.60 (±1.09)	0.67 (±1.24)
Operation Duration (minutes)	18.03 (±11.86)*	11.88 (±5.71)	12.09 (±5.88)
Post-op Questionnaire (Yes/No)			
Question 1	31/0	35/0	30/01
Question 2	30/01	31/4	28/03
Question 3	31/0	35/0	30/01
Question 4	30/01	33/2	30/01

* Statistically significant difference between groups (p<0.05).

**Table 2:** Comparison of hemodynamic parameters at different time points across the groups.

	Group 1			Group 2			Group 3			
T1	All	Female	Male	All	Female	Male	All	Female	Male	p *p<0.05
SBP (mmHg)	125.74 (±15.15)	129.60 (±15.44)	122.12 (±14.42)	121.71 (±16.24)	125.22 (±14.09)	118.00 (±17.92)	123.84 (±12.55)	129.55 (±13.36)	120.70 (±11.2)	.782
DBP (mmHg)	74.84 (±12.59)	76.00 (±10.92)	73.75 (±14.26)	73.83 (±11.47)	74.22 (±11.44)	73.41 (±11.84)	72.58 (±11.01)	71.55 (±13.87)	73.15 (±9.43)	.900
HR (bpm)	92.84 (±17.50)	85.07 (±17.65)	100.13 (±14.31)	90.69 (±18.49)	87.06 (±16.27)	94.53 (±20.37)	89.81 (±15.52)	87.73 (±11.85)	90.95 (±17.4)	.885
SpO2 (%)	98.39 (±1.28)	98.07 (±0.96)	98.69 (±1.49)	98.63 (±2.03)	98.56 (±2.59)	98.71 (±1.26)	98.81	98.82 (±0.98)	98.80 (±1.11)	.770
T2										
SBP (mmHg)	124.84 (±14.41)	129.8 (±15.53)	120.19 (±11.95)	121.09 (±13.47)	122.78 (±12.86)	119.29 (±14.26)	125.39 (±14.52)	132.36 (±13.26)	121.55 (±14.0)	.438
DBP (mmHg)	72.68 (±10.11)	74.47 (±10.08)	71.00 (±10.16)	74.14 (±11.44)	74.50 (±12.71)	73.76 (±10.30)	73.74 (±10.85)	75.91 (±15.40)	72.55 (±7.53)	.553
HR (bpm)	93.84 (±16.71)	86.47 (±13.60)	100.75 (±16.75)	88.80 (±17.43)	87.06 (±17.22)	90.65 (±17.98)	92.84 (±15.36)	91.36 (±16.08)	93.65 (±15.3)	.413
SpO2 (%)	98.26 (±2.44)	98.07 (±1.16)	96.94 (±4.41)	97.86 (±3.33)	96.94 (±4.41)	98.82 (±0.95)	98.90 (±1.01)	99.09 (±0.94)	98.80 (±1.06)	.327
T3										
SBP (mmHg)	121.94 (±14.99)	125.33(±15.91)	118.75 (±13.82)	120.66 (±13.97)	120.22 (±15.74)	121.12 (±12.29)	122.55 (±15.09)	128.27 (±12.53)	119.4 (±15.7)	.873
DBP (mmHg)	69.55 (±8.41)	72.73 (±8.15)	66.56 (±7.74)	71.11 (±11.28)	70.5 (±13.06)	71.76 (±9.39)	74.97 (±9.34)*	79.09 (±11.05)	72.70 (±7.63)	.026*
HR (bpm)	94.61 (±16.98)	92.20 (±16.84)	96.88 (±17.33)	89.86 (±18.10)	85.89 (±18.04)	94.06 (±17.73)	91.84 (±15.69)	90.91 (±18.60)	92.35 (±14.3)	.452
SpO2 (%)	96.55 (±6.48)	97.73 (±1.22)	95.44 (±8.94)	97.86 (±3.67)	96.78 (±4.81)	99.00 (±1.17)	98.77 (±1.23)	98.82 (±1.08)	98.75 (±1.33)	.167
T4										
SBP (mmHg)	126.19 (±14.62)	130.33 (±18.53)	122.31 (±8.60)	118.86 (±18.00)	120.22 (±22.31)	117.41 (±12.48)	125.19 (±16.5)	130.45 (±15.89)	122.3 (±16.5)	.115
DBP (mmHg)	72.42 (±10.38)	74.27 (±8.46)	70.69 (±11.91)	75.09 (±20.13)	72.61 (±15.59)	77.71 (±24.26)	72.97 (±8.52)	76.09 (±7.44)	71.25 (±8.77)	.655
HR (bpm)	94.58 (±15.50)	89.33 (±12.58)	99.50 (±16.72)	89.09 (±18.69)	85.89 (±18.82)	92.47 (±18.51)	93.23 (±13.8)	91.27 (±13.29)	94.30 (±14.2)	.237
SpO2 (%)	98.23 (±2.06)	98.27 (±1.16)	98.19 (±2.69)	98.11 (±1.28)	97.61 (±1.2)	98.65 (±1.17)	98.94 (±0.89)*	99.03 (±1.00)	98.90 (±0.85)	.041*
T5										
SBP (mmHg)	123.71 (±12.62)	128.47 (±14.61)	119.25 (±8.69)	117.80 (±14.28)	116.28 (±15.25)	119.41 (±13.44)	119.03 (±14.76)	124.27 (±16.88)	116.15 (±13.0)	.215
DBP (mmHg)	72.81 (±10.81)	75.13 (±7.79)	70.63 (±12.91)	70.57 (±12.74)	69.67 (±15.79)	71.53 (±8.86)	71.52 (±8.72)	72.45 (±10.98)	71.00 (±7.48)	.768
HR (bpm)	91.29 (±15.93)	85.93 (±15.15)	96.31 (±15.42)	83.54 (±14.13)	81.56 (±15.86)	85.65 (±12.17)	86.39 (±13.52)	86.09 (±13.08)	86.55 (±14.0)	.124
SpO2 (%)	98.26 (±1.26)	98.13 (±0.99)	98.38 (±1.50)	97.89 (±1.78)	97.39 (±1.69)	98.41 (±1.77)	98.87 (±1.09)*	98.91 (±1.30)	98.85 (±0.99)	.018*

*Significant differences were observed in SpO2 (T4), SpO2 (T5) and DBP (T3).

**Table 3:** Table Preoperative and postoperative anxiety scores measured by MDAS and STAI in control, verbal and visual information groups.

	MDAS		STAI-T		STAI-S	
Groups	Pre-op	Post-op	Pre-op	Post-op	Pre-op	Post-op
Group 1	11.48(±4.28) a	8.77(±3.04)	43.77(±8.70) a	34.26(±8.58)	40.06(±7.75)	39.56(±7.64)
Female	9.13(±3.81)*	7.20(±2.51)	39.93(±9.14)*	31.13(±8.85)	37.20(±6.48)	36.6(±8.11)
Male	13.69(±3.52)*	10.25(±2.79)	47.38(±6.69)*	37.19(±7.44)	42.75(±8.05)*	40.53(±8.15)
Group 2	11.80(±4.79)	10.80(±5.36)	40.94(±9.98) a	35.89(±11.43)	39.97(±7.69)	38.71(±8.26)
Female	10.22(±4.01)	10.00(±5.62)	37.61(±8.67)	34.33(±10.87)	40.00(±7.13)	38.56(±7.79)
Male	13.47(±5.09)*	11.65(±5.11)	44.47(±10.30)*	37.53(±12.10)	39.94(±8.47)	38.88(±8.97)
Group 3	11.97(±4.46) a	9.74(±3.93)	41.10(±9.03) a	36.19(±11.17)	39.16(±7.96)	38.52(±6.95)
Female	9.64(±4.48)*	7.64(±3.47)	37.00(±9.36)	33.27(±10.98)	37.64(±6.02)	37.45(±7.09)
Male	13.25(±4.00)*	10.90(±3.75)	43.35(±8.22)*	37.80(±11.22)	39.10(±6.98)	37.80(±11.22)

*: Significantly (p<0.05) higher between pre-op and post-op values in gender specific subgroups. a: Significantly (p<0.05) higher between pre-op and post-op values in main groups. Pre-op: Preoperative. Post-op: Postoperative. MDAS: Modified Dental Anxiety Scale. STAI: The State-Trait Anxiety Inventory.

## Data Availability

The datasets used and/or analyzed in this study are available from the corresponding author on reasonable request.
